# The topology of chromatin-binding domains in the NuRD deacetylase complex

**DOI:** 10.1093/nar/gkaa1121

**Published:** 2020-12-02

**Authors:** Christopher J Millard, Louise Fairall, Timothy J Ragan, Christos G Savva, John W R Schwabe

**Affiliations:** The Leicester Institute of Structural and Chemical Biology, Department of Molecular and Cell Biology, University of Leicester, Leicester LE1 7RH, UK; The Leicester Institute of Structural and Chemical Biology, Department of Molecular and Cell Biology, University of Leicester, Leicester LE1 7RH, UK; The Leicester Institute of Structural and Chemical Biology, Department of Molecular and Cell Biology, University of Leicester, Leicester LE1 7RH, UK; The Leicester Institute of Structural and Chemical Biology, Department of Molecular and Cell Biology, University of Leicester, Leicester LE1 7RH, UK; The Leicester Institute of Structural and Chemical Biology, Department of Molecular and Cell Biology, University of Leicester, Leicester LE1 7RH, UK

## Abstract

Class I histone deacetylase complexes play essential roles in many nuclear processes. Whilst they contain a common catalytic subunit, they have diverse modes of action determined by associated factors in the distinct complexes. The deacetylase module from the NuRD complex contains three protein domains that control the recruitment of chromatin to the deacetylase enzyme, HDAC1/2. Using biochemical approaches and cryo-electron microscopy, we have determined how three chromatin-binding domains (MTA1-BAH, MBD2/3 and RBBP4/7) are assembled in relation to the core complex so as to facilitate interaction of the complex with the genome. We observe a striking arrangement of the BAH domains suggesting a potential mechanism for binding to di-nucleosomes. We also find that the WD40 domains from RBBP4 are linked to the core with surprising flexibility that is likely important for chromatin engagement. A single MBD2 protein binds asymmetrically to the dimerisation interface of the complex. This symmetry mismatch explains the stoichiometry of the complex. Finally, our structures suggest how the holo-NuRD might assemble on a di-nucleosome substrate.

## INTRODUCTION

The Nucleosome Remodelling and Deacetylase (NuRD) complex is a multi-protein chromatin modifying machine that plays an essential role in cell lineage commitment and DNA damage repair (1,2). The NuRD complex localises to both gene promoters / enhancers and sites within gene bodies (3,4). The complex controls expression of genes through two distinct activities: removal of acetyl groups from chromatin through the action of a histone deacetylase enzyme and remodelling of nucleosomes through an ATP-dependent helicase. These two activities control genome packaging, gene expression and ultimately cellular identity.

The NuRD complex contains seven core components, each of which has multiple paralogues, several with distinct expression patterns. This results in considerable subunit variability (5,6). The core components are histone deacetylases 1 and 2 (HDAC1/2), metastasis associated scaffold proteins (MTA1/2/3), histone chaperones (RBBP4/7), methyl-DNA binding domain proteins (MBD2/3), GATA-type zinc finger proteins (GATAD2a/b), the ATP-dependent helicases (CHD3/4/5) and cyclin dependent kinase associated protein (CDK2AP1) (for reviews, see (7,8)). In addition to the core components there are a number of more transient members of the complex including FOG1, BCL11A/B and SALL1 which associate with the NuRD complex to recruit the dual enzymatic activity to specific gene loci (9,10).

It has recently emerged that a significant fraction of the NuRD complex lacks the remodelling components (GATA2Da/b, CHD3/4/5, CDK2AP1) and contains just the deacetylase module (HDAC1/2, MTA1/2/3, RBBP4/7) (11). This deacetylase module has been shown to be a fully active and is one of several intermediate and alternative NuRD complexes that have been shown to exist in wild type cells (6,12). Whether or not the chromatin remodelling module is associated with the deacetylase module seems to depend on which of the mutually exclusive components PWWP2A or MBD2/3 is bound to the core deacetylase complex (4).

Within the deacetylase module of NuRD several proteins/domains contribute to chromatin interaction. By analogy with other BAH domains, the BAH domain of MTA1 is thought to bind to nucleosomes (13,14). The WD40 domain proteins RBBP4/7 have been shown to direct binding to the amino-terminal tail of histone H3 (15–17). MBD2/3 also targets the NuRD complex to chromatin (18,19). Interestingly, the paralogues, MBD2 and MBD3, are mutually exclusive components of the core complex that have been demonstrated to have strikingly different biological roles (20). Both have DNA-targeting properties, and whereas MBD2 preferentially binds to methylated and hemi-methylated DNA, MBD3 has a much-reduced preference for methylated sites and binding to DNA is significantly weaker (21,22). Both MBD proteins consist of a DNA-binding domain, an intrinsically disordered region (IDR), and a coiled-coil that binds to GATA2a/b (23). As mentioned above, MBD2/3 can be replaced by PWWP2A forming a deacetylase complex lacking the remodelling module. PWWP2A/B also has chromatin binding activity recognising histone H3 K36me3 (4).

The stoichiometry of assembly of the NuRD complex has been somewhat controversial. Mass spectrometry studies have reported a variety of different stoichiometries for the NuRD complex (11,24–27). Some of the variability may derive from the purification method used to obtain a homogeneous sample. This uncertainty was resolved in part through structural studies of the MTA1:HDAC1 complex which clearly showed that MTA1 forms a dimeric scaffold recruiting two copies of HDAC1 (28). Further structural studies revealed that each MTA1 protein is able to recruit two copies of RBBP4 confirming an overall subunit ratio of 2:2:4 (HDAC1:MTA1:RBBP4) (15,29). The question of how many copies of MBD2/3 are assembled into the NuRD complex has been more challenging. Mass spectrometry suggests that this component may be sub-stoichiometric to the others and that only a single MBD2/3 is recruited to a NuRD:MTA1 dimer (24).

We have investigated the architecture of the NuRD deacetylase module to understand the stoichiometry and topological arrangement of the chromatin binding modules with a view to understanding how the complex interacts with chromatin. We demonstrate unambiguously that a single copy of MBD2 binds to the HDAC1:MTA1 dimerisation interface and indeed binding requires a pre-formed dimeric complex. We use cryo-electron microscopy (cryo-EM) to visualise the architecture of three progressively larger NuRD complexes. These structures reveal how the BAH domains of MTA1, the RBBP4/7 WD40 domains and the MBD2 methyl-DNA binding domain are positioned relative to each other and the HDAC1 dimer. These findings suggest how the complex is targeted to chromatin.

## MATERIALS AND METHODS

### Mammalian protein expression and purification

Wild type and mutant MTA1 constructs were cloned into pcDNA3 vectors containing an amino terminal His_10_-Flag_3_ purification tag and a TEV protease cleavage site. Full length HDAC1 (residues 1–482), RBBP4 (residues 1–425) and MBD2 (residues 145–411) were cloned without affinity tags into the same vectors. Protein was expressed in HEK293F suspension-grown cells (Invitrogen) using polyethylenimine (PEI; Sigma) as a transfection reagent and harvested after 48 h as previously described (15,30). Cells were lysed in 50 mM Tris–HCl (pH 7.5), 100 mM potassium acetate, 10% (v/v) glycerol, 0.3% (v/v) Triton X-100 and Roche Protease Inhibitor (buffer A). The lysate was clarified by centrifugation and applied to FLAG resin (Sigma) for 30 min and washed three times with 50 mM Tris–HCl (pH 7.5), 100 mM potassium acetate and 5% (v/v) glycerol (buffer B). The protein was treated with RNase A in buffer B for 1 h, washed twice more with 25 mM Tris–HCl (pH 7.5), 75 mM potassium acetate and 0.5 mM TCEP (buffer C), before being eluted with TEV protease overnight. The protein complexes were purified further by gel filtration using a Superose 6 column (GE Healthcare, UK).

### Strep-tag purification

MBD2 (residues 114–411) with 8 residue non-cleavable strep tag was cloned into a pcDNA vector (Strep-MBD2). Strep-MBD2 and untagged MBD2 were co-expressed with MTA1 and HDAC1 in HEK293F cells and the complex containing FLAG-MTA1:HDAC1:MBD2/Strep-MBD2 was purified on FLAG-resin as detailed above. The purified complex was eluted with TEV protease overnight, gel filtrated using a Superose 6 column and applied to Strep-Tactin XT Superflow resin (IBA Lifesciences). The Strep-resin was washed five times with buffer C before being directly loaded onto the SDS-PAGE gel.

### Size exclusion chromatography with multi-angle light scattering (SEC-MALS)

Gel filtration-pure HDAC1:MTA1:MBD2 complex was analysed by SEC-MALS. The peak eluted fraction from a Superose 6 column was re-run down the same column and monitored with an Optilab T-rEX differential Refractive Index detector coupled to a DAWN HELEOS MALS detector (Wyatt Technology). The mass of each protein complex was calculated using ASTRA software version 6.1.

### EM sample preparation and data collection

MTA1_(ELM2-SANT)_, MTA1_(BAH-ELM2-SANT-ZnF)_ and MTA1_(BAH-ELM2-SANT-ZnF-R1)_ complexes (containing HDAC1, MBD2, RBBP4) were purified by gel filtration and applied to Quantifoil R1.2/1.3 grids overlaid with graphene oxide, as described in (31). Grids were blotted and plunge frozen using a Vitrobot 4. Data for the MTA1_(ELM2-SANT)_ and MTA1_(BAH-ELM2-SANT-ZnF-R1)_ complexes were collected on an FEI Titan Krios operating at an accelerating voltage of 300 kV equipped with an FEI Falcon 3 direct electron detector in counting mode using a phase plate. Micrographs were collected at a nominal magnification of ×75K (calibrated pixel size of 1.08 Å) and a dose rate of 0.68e^−^/pixel/s in counting mode over 60 s and 75 fractions. Data for the MTA1_(BAH-ELM2-SANT-ZnF)_ complex was collected using a Gatan K3 direct electron detector at super-resolution mode. Micrographs were collected at a nominal magnification of ×81K (calibrated pixel size of 1.09 Å) and a dose rate of 10e^−^/pixel/sec over 5 s and 45 fractions. Conventionally defocused and phase plate datasets were collected using the EPU software (Thermo Fisher Scientific). Micrographs were collected at various defocus modes between 0.5 and 3.5 μm.

### EM image processing

All datasets were processed in Relion3.0 (32). Micrographs were first corrected for large movements using MotionCorr2 (33) and CTF parameters were estimated using GTCF (34). Autopicking was performed in Relion using the LOG autopicking software. The resolution was calculated using Gold-Standard 0.143 criterion, resulting in 6.1 and 19 Å for the two Falcon 3 datasets (MTA1_(ELM2-SANT)_ and MTA1_(BAH-ELM2-SANT-ZnF-R1)_ complexes) and a 4.5 Å map for the K3 dataset (MTA1_(BAH-ELM2-SANT-ZnF)_ complex). Box sizes of 150, 210 and 280 pixels were used for the MTA1_(ELM2-SANT)_, MTA1_(BAH-ELM2-SANT-ZnF)_ and MTA1_(BAH-ELM2-SANT-ZnF-R1)_ complexes respectively. The NMR solution structure of MBD2 (pdb code: 2KY8) and crystal structures of HDAC1:MTA1 and RBBP4:MTA1 (pdb codes: 5ICN and 5FXY) were docked as rigid bodies in Chimera (35). Phenix validation for CryoEM was used to calculate the FSC model-to-map (36). Image processing statistics are shown in Supplementary Table S1.

### DNA binding assay

Binding of the HDAC1:MTA1:MBD2 to DNA was tested on a short fragment of 15 nt DNA sequence GGAATmCGGCTCATGC by electrophoretic mobility shift assay. The probe mixture of double stranded DNA was prepared by annealing complementary oligonucleotides as described in (37). Briefly, 1 μM double stranded DNA was incubated with a 1- to 10-fold excess of complex in binding buffer (20 mM HEPES pH 7.0, 75 mM potassium acetate and 2 mM DTT) for 20 min on ice. Samples were analysed on a 10 cm × 10 cm, 5% acrylamide gel buffered in 0.5× TB (45 mM Tris, 45 mM boric acid). The gel was stained with ethidium bromide and visualized using UV.

## RESULTS

### MBD2 interacts directly with the HDAC1:MTA1 dimer

**Figure 1.**
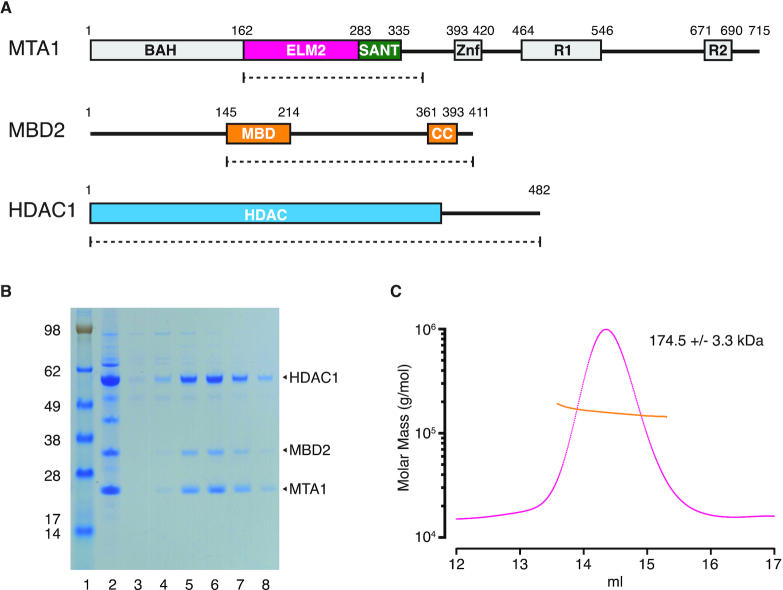
MTA1 and HDAC1 copurify with MBD2. (**A**) Schematic of the domain structure of MTA1, MBD2 and HDAC1. The fragments used in the interaction study are shown underneath as dotted lines. (**B**) Gel filtration of the MTA1 (residues 162–354), HDAC1 and MBD2 (145–411) complex on a Superose 6 column. The complex before column purification is shown in lane 2 and fractions from the column in lanes 3–8. (**C**) SEC-MALS profile for the MTA1 (residues 162–354), HDAC1 and MBD2 (145–411) complex. The measured molecular weight is 174.5 kDa and the calculated molecular weight for a stoichiometry of 2:2:1 is 184.0 kDa.

To resolve the question of the stoichiometry of MBD2 in the NuRD complex we expressed a flag-tagged construct of MTA1 containing the ELM2-SANT domain (residues 162–354) with HDAC1 and MBD2 and purified the complex on anti-flag resin. The complex was washed, cleaved from the resin by TEV protease, and further purified by gel filtration. As expected, MBD2 co-purified as part of the complex but appeared to be sub-stoichiometric relative to HDAC1 and MTA1 as judged by the intensity of bands on an SDS PAGE gel (Figure 1A and B). To eliminate the possibility of low MBD2 protein expression we transfected cells at a 1:1:1 DNA ratio and also with a four-fold excess of MBD2 DNA (1:1:4) but observed the same apparently sub-stoichiometric ratio in both cases (Supplementary Figure S1).

Since we had previously shown that two molecules of HDAC1 bind to dimeric MTA1 (28) the weaker intensity of the MBD2 band could either be due to poor uptake of the coomassie stain or more interestingly by a sub-stoichiometric ratio. Therefore, we investigated the stoichiometry of the complex using size exclusion chromatography followed by multi-angle light scattering (SEC-MALS). We expressed a construct of MBD2 which was missing the disordered N-terminus (residues 145–411). The average molecular weight of the HDAC1:MTA1:MBD2 complex was determined to be 174.5 kDa which fits well with the predicted molecular weight (184kDa) of a dimeric HDAC1:MTA1 bound to a single MBD2, i.e. indicating a stoichiometry of 2:2:1 (Figure 1C).

### A single copy of MBD2 is recruited to a HDAC1:MTA1 dimer complex

**Figure 2.**
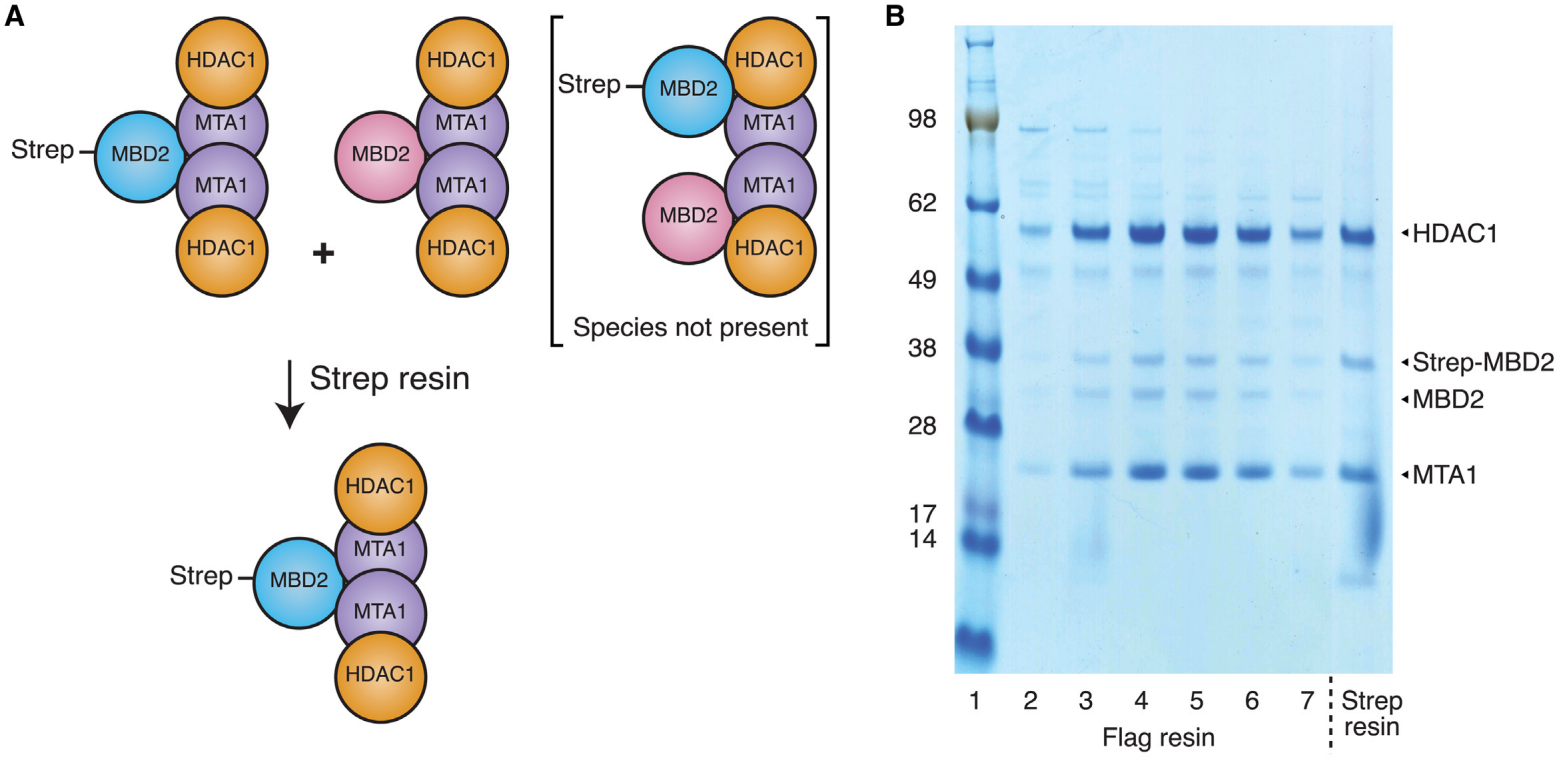
The HDAC1:MTA1 dimer only binds to a single copy of MBD2. (**A**) Schematic of the binding experiment outlining two possible scenarios. (**B**) Sequential flag and strep purifications of the complex. The flag-tagged complex was cleaved from the resin and purified on a Superose 6 gel filtration column (lanes 2–7). Lane 5 complex was re-applied to Strep-resin and washed before loading on the gel.

To confirm the stoichiometry of the HDAC1:MTA1:MBD2 complex we designed two different length constructs of MBD2 that could be distinguished by SDS-PAGE; one with a strep-tag and one without, and co-expressed these with HDAC1 and MTA1. Our two-step strategy was to purify these MBD2 constructs on anti-flag resin (using the flag tag on MTA1 as bait) before further purifying the complex on strep resin (Figure 2A). If the untagged MBD2 protein remained in the complex following the strep purification step, this would suggest a 2:2:2 stoichiometry, but a single MBD2 band would indicate a 2:2:1 complex. The SDS-PAGE gel showed that while both MBD2 constructs were present on the flag resin, the untagged MBD2 material was lost on the strep resin (Figure 2B). This confirms the conclusion that a single MBD2 polypeptide binds to the HDAC1:MTA1 dimer.

Further support for a 2:2:1 HDAC1:MTA1:MBD2 ratio was provided by an EMSA DNA-binding assay with a methylated DNA-duplex. Only a single bound species was observed (Supplementary Figure S1c). If two MBD2 proteins were bound then the HDAC1:MTA1:MBD2 complex would be expected to bind to one or two DNA duplexes depending upon the DNA:complex ratio.

### The HDAC1:MTA1 dimerization interface forms part of the MBD2 binding site

We reasoned that since a single MBD2 binds to the HDAC1:MTA1 dimer, MBD2 must bind in such a way as to sterically occlude a second molecule binding. Initially we attempted to express shortened constructs of MBD2 to define the minimal region required for interaction. However, expression constructs trimmed shorter than residues 145–393 did not co-purify with the complex. This suggests that these shorter constructs either bind less tightly and are lost during purification or that the whole region 145–393 is essential for interaction. To explore this further, we made structure-guided mutations of the MTA1 dimerisation interface to probe whether changes in this region might perturb binding of MBD2.

**Figure 3.**
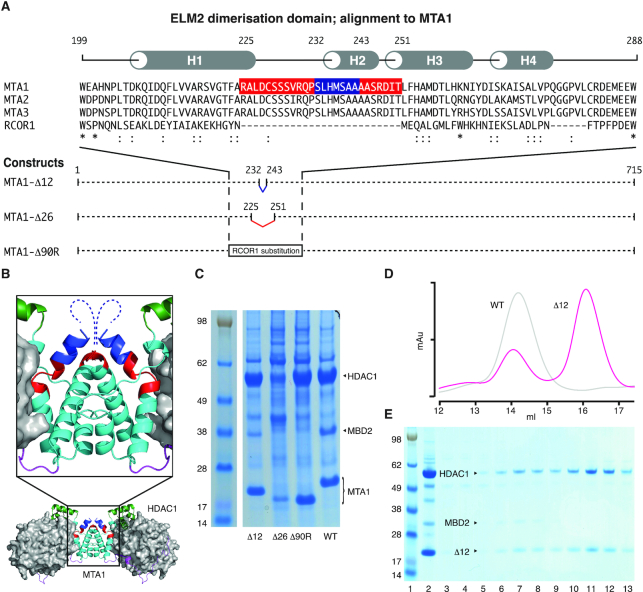
The MTA1 dimerisation interface is important for MBD2 recruitment. (**A**) Sequence alignment of ELM2 domains from MTA1, MTA2, MTA3 and RCOR1. The secondary structure of MTA1 is indicated above the sequences. Three MTA1 deletion constructs are shown below the alignment. The 90 residues deleted from MTA1 in the MTA1-Δ90R construct are substituted by the corresponding residues from RCOR1. (**B**) The crystal structure of the HDAC1:MTA1 dimer (pdb code: 5ICN, (39)) highlighting the deletions on the structure: blue residues are deleted in MTA1-Δ12; blue and red deleted in MTA1-Δ26; blue, red and cyan are deleted and replaced in MTA1-Δ90R. (**C**) SDS-PAGE gel showing the proteins pulled down by mutant and wildtype MTA1 constructs. (**D**) Gel filtration profile on a Superose 6 column of the MTA1-Δ12 mutant complex (pink) compared with MTA1 wildtype (grey). (**E**) SDS-PAGE of the Superose 6 gel filtrated MTA1-Δ12 complex. Complex before the column is shown in lane 2 and the fractions from the column in lanes 3–13. The peak fractions are lanes 7 and 11.

The homo-dimerisation interface of MTA1 is well characterised and consists of several helices from the ELM2 domain of each MTA1 monomer coming together to form a hydrophobic core (15,28). We created two deletion mutants and a chimeric insertion within the ELM2 domain of MTA1 (162–354) (Figure 3A and B). The first deletion construct had 12 residues missing to remove part of the ELM2 helix 2 (MTA1-Δ12). The second deletion construct was based on alignment with RCOR1 and the whole of helix 2 (MTA1-Δ26) was deleted. The third mutant (MTA1-Δ90R) was a chimeric protein in which residues 199–288 were substituted with the equivalent residues from RCOR1, a monomeric distant homologue of MTA1 (38).

Co-transfection and purification of the MTA1 mutants with HDAC1 and MBD2 showed that all three mutant proteins were expressed and able to form a complex with HDAC1. However, both the MTA1-Δ26 and MTA1-Δ90R complexes failed to interact with MBD2 (Figure 3c). Interestingly the MTA1-Δ12 complex (i.e. the smallest deletion) retained ability to interact with MBD2 (Figure 3C). However, MBD2 recruitment was reduced compared with wild type, suggesting that the MBD2 binding site had been weakened.

To understand why the recruitment of MBD2 was reduced in the MTA1-Δ12:HDAC1:MBD2 mutant complex, we scaled up expression and purified the complex on a size exclusion column. The chromatogram showed two peaks: a larger dimeric species that matched the wild type complex, and a smaller complex that did not contain MBD2 (Figure 3D and E). This second peak appears to be the monomeric form of the complex since the elution point is characteristic of a 70–80 kDa complex. This suggests the mutation weakens, but does not abrogate dimerisation of the complex. Interestingly, the dimeric complex, but not the monomeric, retains the ability to bind MBD2 despite containing the mutation, suggesting that the deleted surface is itself not important for binding (Figure 3E). This strongly suggests that dimerisation is required for MBD2 binding. Both the MTA1-Δ26 and MTA1-Δ90R complexes fail to bind MBD2. This is not unexpected since they are both monomeric. It is also possible that key interactions are perturbed by these mutations.

### The structure of MBD2 bound to the HDAC1:MTA1 dimer

**Figure 4.**
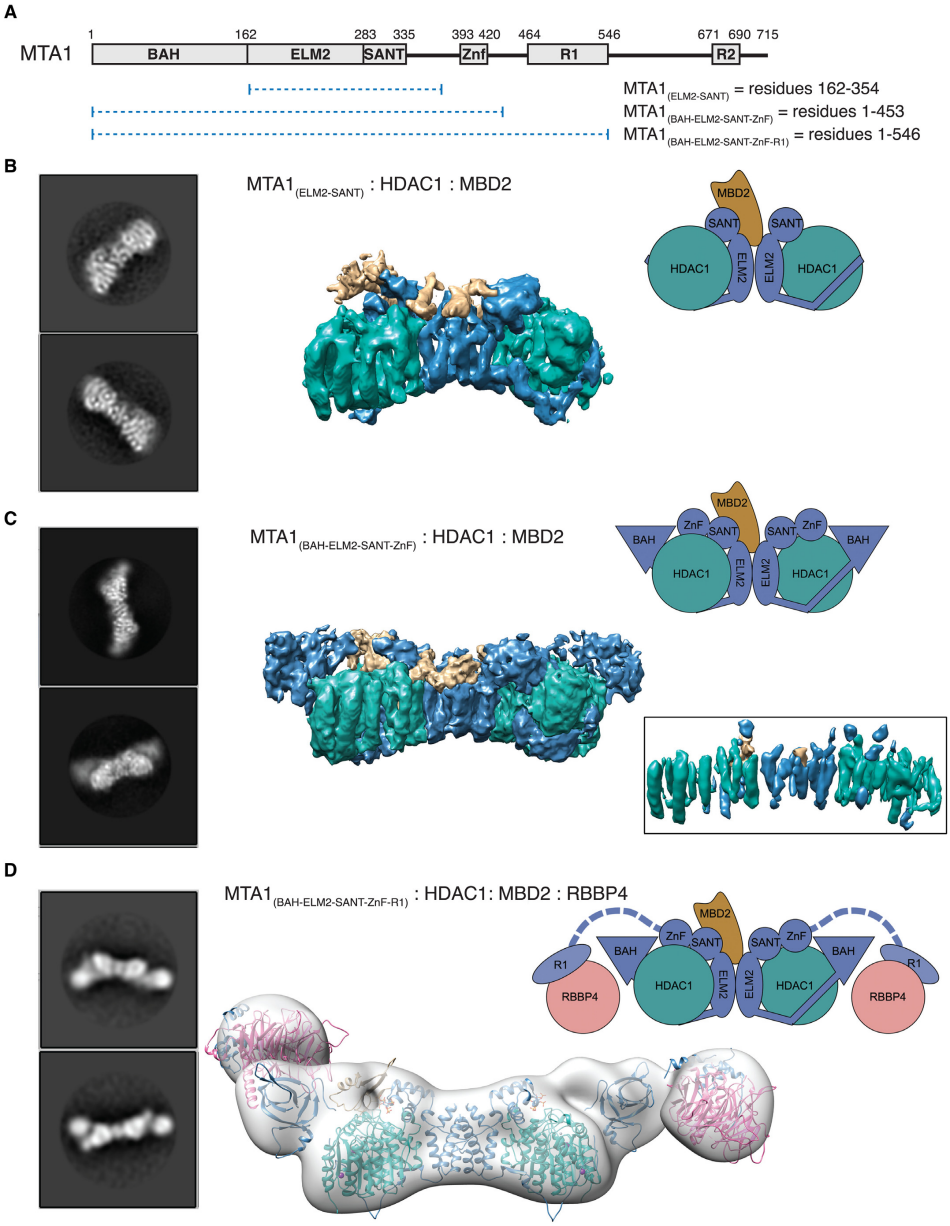
Visualisation of the core NuRD complex by cryo electron microscopy. (**A**) Schematic of the MTA1 constructs (shown as dotted lines) used to recruit HDAC1, MBD2 and RBBP4 to the NuRD complex. (**B**) Representative 2D class averages and 3D map of the complex containing MTA1 (ELM2 and SANT domains; residues 162–354), HDAC1 and MBD2 (145–411). A cartoon representation is shown to highlight the arrangement of subunits. HDAC1, MTA1 and MBD2 are coloured green, blue and tan respectively. (**C**) Representative 2D class averages and 3D map of the complex containing an extended MTA1 construct (BAH, ELM2, SANT and ZnF domains; residues 1–453), HDAC1 and MBD2 (145–411). Map contour level is 0.01 and inset map contour level is 0.02—Chimera. (**D**) Representative 2D class averages and 3D map of the complex containing a MTA1 construct (BAH, ELM2, SANT, ZnF and R1 domains; residues 1–546), HDAC1 and MBD2 (145–411) to visualise how RBBP4 is recruited to the complex. The crystal structures of HDAC1:MTA1 (5ICN, (39)), RBBP4:MTA1 (5FXY, (15)) and NMR solution structure of MBD2 (2KY8, (22)) are fitted into the map. RBBP4 is shown in pink.

To visualise how MBD2 interacts with the HDAC1:MTA1 dimer we used cryo-electron microscopy to determine the structure of MBD2 bound to the MTA1_(ELM2-SANT)_:HDAC1 complex (Figure 4A). A map was calculated using 70 561 particles to a resolution of 6.1 Å (Figure 4B, Supplementary Figures S2–S4). The dimeric nature of the complex was immediately apparent in the raw micrographs with front-on particles resembling ‘bowties’ (i.e. two HDACs flanking the MTA1 dimer interface). The helices of MTA1 and HDAC1 are clearly visible in the 2D class averages and the crystal structure of the HDAC1:MTA1 dimer (pdb code: 5ICN, (39)) could be readily docked into the 3D refined map using Chimera (Supplementary Figure S2).

On fitting the HDAC1:MTA1 dimer structure into the map, it was apparent that the complex was asymmetric with additional density appearing on one but not both of the MTA1-SANT domains. While the resolution of the map was too low for *de novo* building, the NMR solution structure of the MBD domain of MBD2 (residues 145–210) (pdb code: 2KY8, (22)) could be fitted into this density. Having placed the two crystal structures into the map, the remaining unaccounted-for density was extracted and examined. Particularly interesting was density from the intrinsically disordered domain (IDR) of MBD2, and the CC domain, which appears to wrap around both sides of the dimerisation interface (Supplementary Figure S2). On inspection of the MBD2 sequence we identified a short hydrophobic motif (I/VTxQI/V) that is repeated twice in MBD2 with a linker of 36 amino-acids (Supplementary Figure S2e). This region of MBD2 had been previously shown to be important for binding and indeed has been predicted to form a large contact surface with the core HDAC complex (23). Binding of the two motifs across the MTA1 dimer interface would explain why one bound MBD2 would occlude binding of a second MBD2.

### The BAH domain from MTA1 is ideally positioned to recruit nucleosomes to the active site

The BAH domain in MTA1 is thought to play a role in recruiting nucleosomes to the deacetylase complex. To understand the relationship between the BAH domain and the HDAC catalytic site, we prepared a complex containing an extended construct of MTA1 including the BAH, ELM2, SANT and GATA zinc finger (ZnF) domains (residues 1–453) together with HDAC1 and MBD2. CryoEM data were collected and a map was calculated to 4.5 Å based on 94 041 particles (Figure 4C, Supplementary Figures S2–S5). A direct comparison of this map with that of the MTA1_(ELM2-SANT)_:HDAC1:MBD2 complex shows that the central portion of the map is higher resolution than the first map and that the envelope of the complex extended, with additional density at either end of the HDAC1:MTA1 dimer. This part of the map has a distinctive, relatively narrow volume suggesting a compact but elongated domain which fits well with the known architecture of BAH domains. This part of the map is not high enough resolution to build *de novo*, but it was straightforward to dock a Phyre model of the MTA1-BAH domain, based on previous crystal structures of BAH domains from ORC1 and SIR3 (pdb codes: 6OM3 and 3TU4, (13,14)). As would be expected, the position of the modelled BAH domain is such that the carboxy-terminus is close to the amino-terminal residue of the ELM2 domain. Although the GATA-like zinc-finger was included in this MTA1 construct, it was not possible to assign density for this in the map, and is speculatively positioned in our schematics (Figure 4C and D).

### The RBBP4/7 modules are flexibly linked to facilitate dynamic chromatin binding

To explore the assembly of the larger complex, we used a longer MTA1 construct (residues 1–546) containing the proximal RBBP4/7 recruitment motif (R1). This MTA1 construct does not include the R2 domain and therefore each MTA1 only recruits a single RBBP4 protein. The 2D and 3D classification of the MTA1_(BAH-ELM2-SANT-ZnF-R1)_:HDAC1:MBD2:RBBP4 complex suggested a relatively high degree of flexibility in the structure and a map of only 19Å using 10 066 particles could be achieved (Figure 4D and Supplementary Figure S6). It is apparent that the flexibility occurs at the point that RBBP4 is tethered to the complex (Supplementary Figure S6). Indeed, the linker residues within MTA1 are predicted unstructured (Protparam, (40)). The flexibility is likely to be important for histone binding by RBBP4 in the context of the whole complex, as well as recruitment to specific transcription factors that interact with RBBP4.

## DISCUSSION

Understanding the assembly and mode of action of the NuRD complex has been a long-standing challenge. In this study we have investigated the stoichiometry of the NuRD deacetylase module and visualised three forms of the complex by electron microscopy. Using two independent techniques, immunoprecipitation and SEC-MALS, we showed that MBD2 is sub-stoichiometric to the HDAC1 and MTA1 proteins and is present in a ratio 2:2:1 (HDAC1:MTA1:MBD2).

Using structure-guided mutation we show that MBD2 interacts with the MTA1 dimerisation interface, likely with a tandemly repeated sequence motif. Partial deletion of MTA1 (helix 2; residues 232–243) partially dissociates the dimer and suggests that MBD2 requires dimerisation to support binding. Interestingly, of all the ELM2-SANT domain HDAC corepressors, MTA from the NuRD complex is the only one that forms a dimer and binds to MBD2. Since MBD2 binds across the dimer interface, our results provide an explanation for why MBD2/3 is specific to the NuRD complex.

Using cryo-electron microscopy, we studied the structure of NuRD complex containing MBD2 and observed clear asymmetry in the complex. The 3D map was high enough resolution to model a single MBD domain (MBD2 residues 145–220) in close proximity to one of the MTA1-SANT domains. Additional volume around the dimerisation interface suggests how the MBD C-terminal region interacts with the HDAC1:MTA1 dimer. MBD2 appears to wrap around the dimerisation interface of MTA1, binding to both front and back surfaces, possibly through a repeated motif. This intrinsically disordered region of MBD2 has been previously shown to be important in recruiting the core HDAC complex (23). The key residues (R286 & L287) identified by Desai *et al.*, are close to the first of the short repeated hydrophobic motifs (I/VTxQI/V) in MBD2 (23). Each part of this multi-domain interface would contribute binding energy to complex formation and would explain why our selective MBD2 deletion constructs did not form stable complexes.

**Figure 5.**
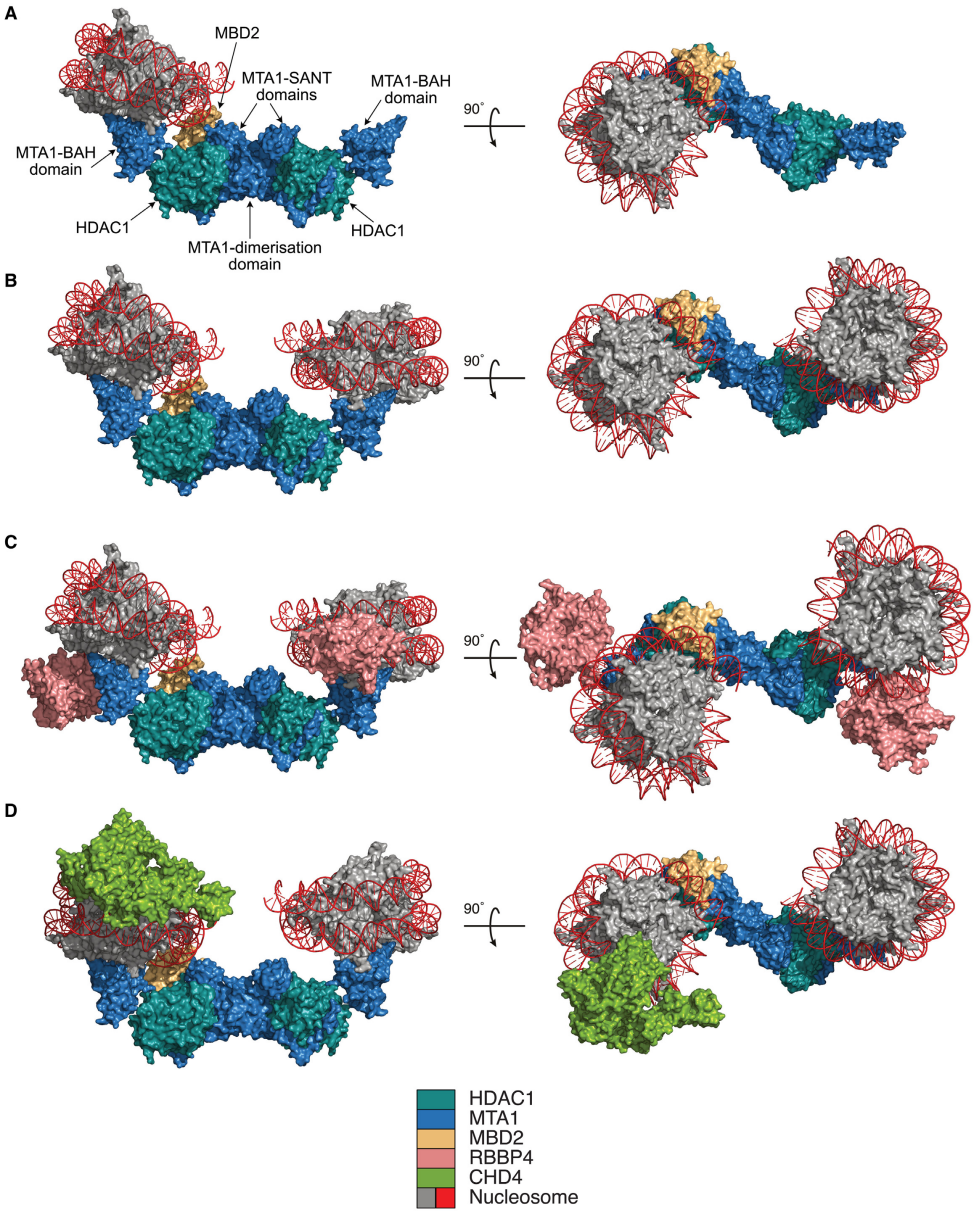
Modelling the recruitment of complexes to chromatin. (**A**) Orthogonal views of a nucleosome from the Sir3:nucleosome crystal structure (3TU4, (13)) superimposed on the MTA1 (BAH-ELM2-SANT-ZnF):HDAC1:MBD2 complex, aligned using the Sir3 BAH domain. (**B**) As in panel (A) with an additional nucleosome docked to the second MTA1-BAH domain, using the Sir3 BAH domain for alignment. (**C**) As in panel (B) with the relative position of the RBBP4-WD40 domains shown in pink. (**D**) Orthogonal views of the CHD4:nucleosome EM structure (6RYR, (43)) superimposed onto the MTA1 (BAH-ELM2-SANT-ZnF):HDAC1:MBD2 complex, aligned using the SIR3 nucleosome position.

Importantly, we were also able to fit the BAH domains of MTA1 into the cryoEM map - even though this part of the map is comparatively less-well defined, likely due to some local motion of the BAH domains. By comparison with the structure of the Sir3 BAH domain bound to a nucleosome (pdb code: 3TU4, (13)), we asked whether this mode of binding was compatible with our structure. Strikingly, the nucleosome would be positioned on the ‘top’ of the structure (Figure 5a) which would bring the histone tails into close proximity to the HDAC active site. Furthermore, the MBD2 DNA-binding domain is in close proximity to the DNA wrapping around the nucleosome and could therefore contribute to binding / recognition - although this would appear to require some structural rearrangement. Finally, although we cannot be sure of the position of the MTA1 zinc finger, carboxy terminal to the SANT domain, it is likely that it is positioned between the SANT and BAH domains. It is therefore also potentially able to interact with the nucleosomal DNA (Figure 5A).

Given that there are two BAH domains in the dimeric complex, it is important to ask whether the NuRD deacetylase module might simultaneously bind to two nucleosomes. Certainly, the structure is compatible with two nucleosomes binding without clashes. However, the relative positioning of the two nucleosomes would suggest that some unwinding of the DNA may be required for a contiguous linker and thus for the two BAH domains to engage with adjacent nucleosomes (Figure 5B). Interestingly, this mode of binding between two nucleosomes is strongly reminiscent of the cryo-EM structure of the Polycomb PRC2 repression complex bound to a di-nucleosome (41).

Although the EM maps suggest that the RBBP4 domains are relatively flexible, their positioning, and apparent range of motion, is broadly compatible with nucleosome binding to the complex (Figure 5C) allowing the WD40 domain to interact with histone H3 amino-termini (16). Interestingly, the flexible attachment of chromatin interaction modules to a deacetylase assembly, was also observed in the MiDAC complex and may be a common feature of histone deacetylase complexes allowing these complexes to adjust to different nucleosome conformations or spacings (42).

Here we have studied the deacetylase module of the NuRD complex. This complex will resemble variants of the NuRD complex that lack the remodelling module (6,11). It is however, important to ask whether this predicted mode of chromatin binding is compatible with the holo-NuRD complex containing the chromatin remodelling module. A recent cryoEM structure of CHD4 bound to a nucleosome allows us to create a composite model of chromatin binding by the two modules (43). In this model the CHD4 and MTA1:HDAC1:MBD2 bind to different surfaces of the nucleosome (Figure 5D). Previous studies have shown that the CHD3/4 remodelling components are recruited to NuRD via a coiled-coil interaction between MBD2 and GATAD2a/b (24,44). Apart from a GATA-type zinc-finger and the coiled-coil MBD2 interaction domain, GATAD2a/b is predicted to be largely disordered and is sufficiently large to bridge the two modules.

It is interesting to speculate reasons why this complex has evolved to bind, asymmetrically, a single MBD protein. One reason could be to maintain MBD2:NuRD, MBD3:NuRD and PWWP2A:NuRD as mutually exclusive complexes acting on different chromatin substrates at different genomic locations. The asymmetry also ensures that only a single CHD3/4 remodeller is associated with the deacetylase module, which may be important for the function of the complex.

## DATA AVAILABILITY

The EM maps for the three parts of the NuRD complex are available from the EMBD under the accession codes EMD-11837, EMD-11838 and EMD-11839. The coordinates for the three models are available from the PDB under the accession codes 7AO8, 7AO9 and 7AOA. All other data are available from the corresponding authors on request.

## Supplementary Material

gkaa1121_Supplemental_File
