# Supplementary material for: The topology of chromatin-binding domains in the NuRD deacetylase complex
Source: Nucleic Acids Res. 2020 Dec 2;48(22):12972–82. doi: 10.1093/nar/gkaa1121 (PMC7736783; doi:10.1093/nar/gkaa1121)
Supplement: gkaa1121_Supplemental_File [file gkaa1121_supplemental_file.pdf]

SUPPLEMENTARY MATERIAL

The topology of chromatin-binding domains in the NuRD deacetylase complex

Christopher J. Millard[+], Louise Fairall, T.J. Ragan, Christos G. Savva and John W.R. Schwabe[+]

The Leicester Institute of Structural and Chemical Biology, Department of Molecular and Cell Biology, University of Leicester, LE1 7RH, UK

[+] Corresponding authors (Email: cm764@le.ac.uk, john.schwabe@le.ac.uk)

Complex	MTA1$_{(ELM2-SANT)}$: HDAC1:MBD2	MTA1$_{(BAH-ELM2-SANT_ZnF)}$: HDAC1:MBD2	MTA1$_{(BAH-ELM2-SANT_ZnF-R1)}$: HDAC1:MBD2:RBBP4
**EMDB ID**	EMD-11838	EMD-11837	EMD-11839
**PDB ID**	7AO9	7AO8	7AOA
**Data collection and processing**			
Magnification	75,000	81,000	75,000
Voltage (kV)	300	300	300
Detector	Falcon 3	K3	Falcon 3
Electron exposure (e^-/pix)	40.8	50.0	40.8
Defocus (μm)	-0.5 (phase plate)	-1.5 to -3.5	-0.5 (phase plate)
Pixel size (Å)	1.08	1.09	1.08
Symmetry imposed	C1	C1	C1
Initial particle images (no.)	515,740	324,135	277,832
Final particle images (no.)	117,161	94,041	10,066
Map resolution (Å)	6.1	4.5	19.4
FSC threshold	0.143	0.143	0.143
FSC model (0.143) *	6.1	5.8	NA
Fitted models (PDB)	4BKX, 2KY8	4BKX, 2KY8	4BKX, 2KY8, 5FXY

Supplementary Table 1. Cryo-EM data collection statistics for the three models of the NuRD deacetylase module. * The FSC model to map was calculated using Phenix validation for CryoEM.

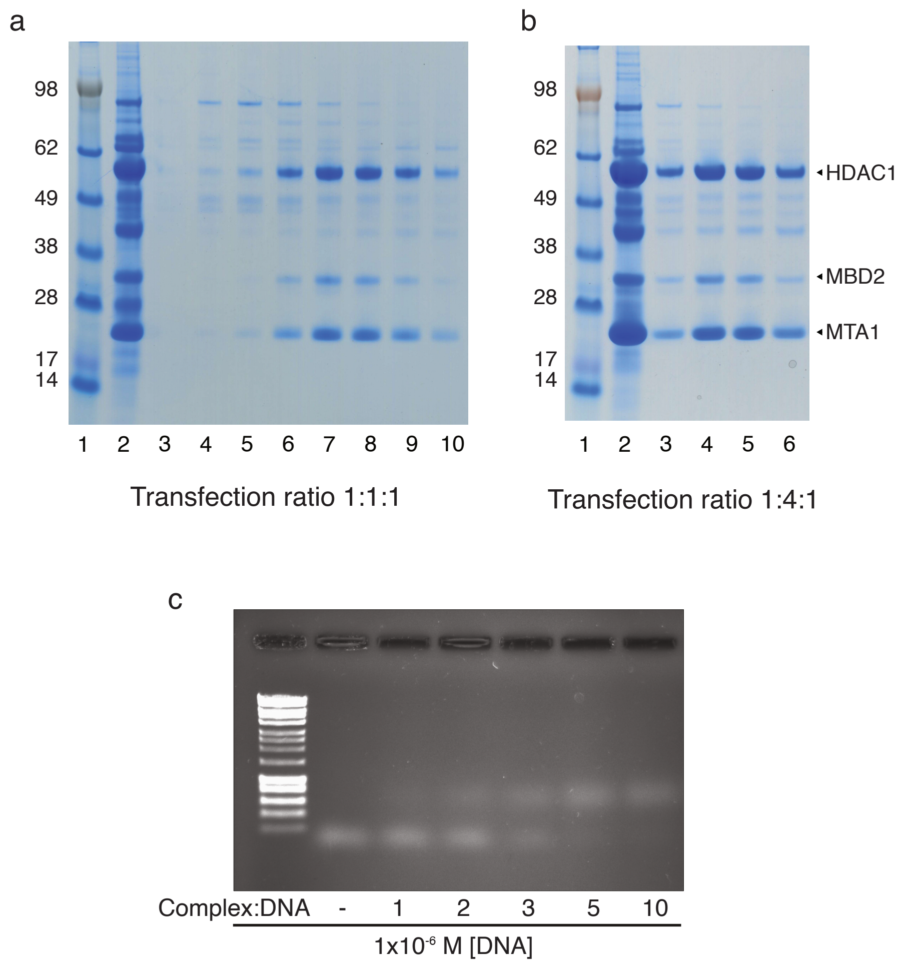

Supplementary Figure S1. The stoichiometry of the complex is consistent across transfections. (a) SDS-PAGE gel showing the purification of HDAC1:MTA1:MBD2 by gel filtration on a Superose 6 column using a DNA transfection ratio of 1:1:1. The complex before the column is shown in lane 2 and fractions from the column in lanes 3-10. (b) Gel filtration of the complex expressed using a DNA transfection ratio of 1:4:1. The complex before the column is shown in lane 2 and fractions from the column in lanes 3-6. (c) EMSA assay of the complex containing MTA1 (residues 1-453), HDAC1 and MBD2 binding to double stranded methylated DNA.

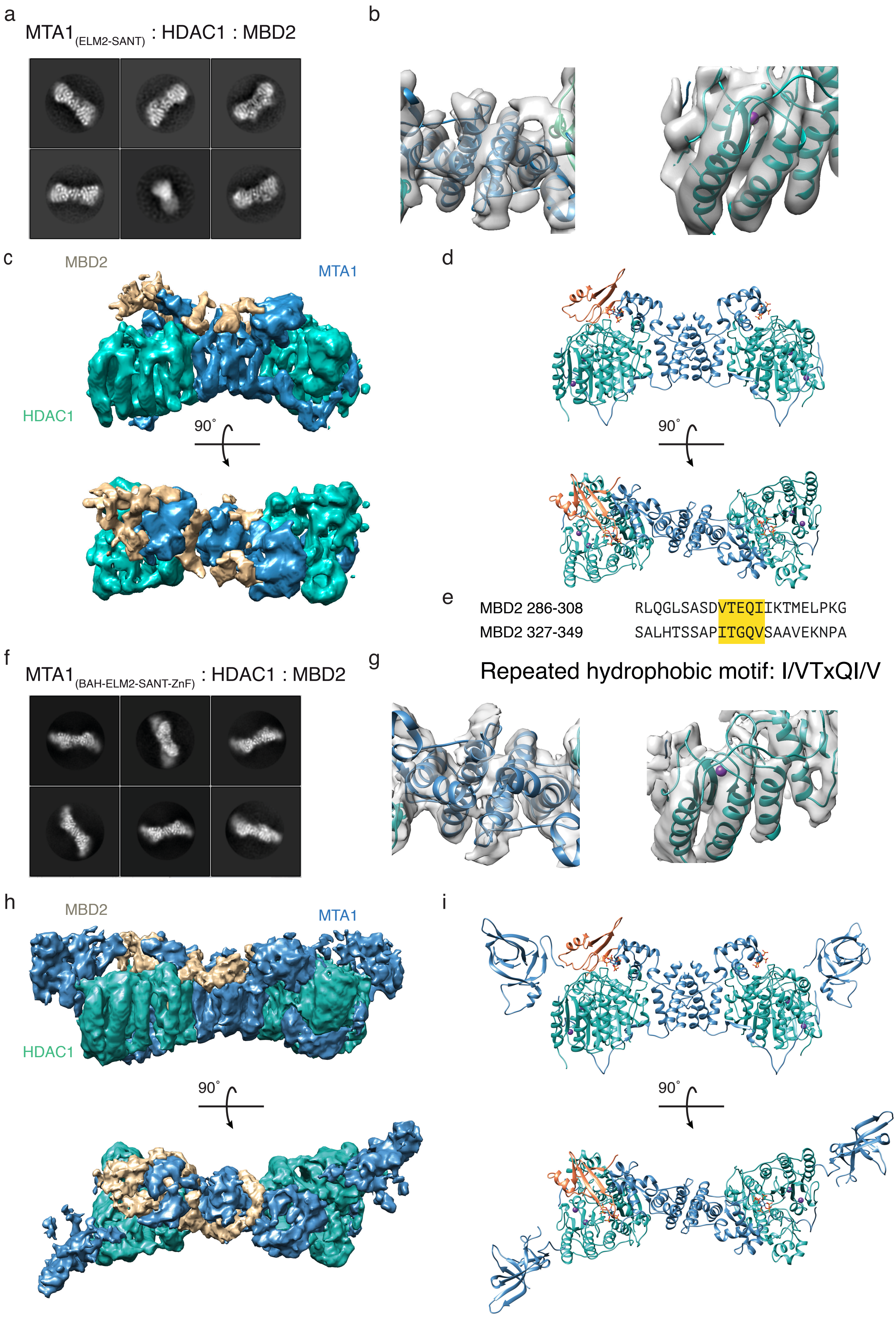

Supplementary Figure S2. Detailed views of NuRD complexes containing HDAC1, MTA1 and MBD2. (a) Additional views of the reference-free 2D class averages of the complex containing MTA1 (residues 162-354), HDAC1 and MBD2. (b) Close-up section of the map with the HDAC1:MTA1 crystal structure (pdb code: 5ICN) fitted into the electron density. (c) Final 3D map of the complex after refinement against 117,161 particles. (d) Model showing the arrangement of the subunits. (e) Putative tandem repeat within MBD2 that may direct pseudo-symmetrical binding to the MTA1 dimerisation interface. (f) Reference-free 2D class averages of the complex containing MTA1 (residues 1-453), HDAC1 and MBD2. (g) Close-up section of the map and HDAC1:MTA1 crystal structure (pdb code: 5ICN). (h) Final 3D map of the complex after refinement against 94,041 particles. (i) Model showing the arrangement of the subunits. HDAC1, MTA1 and MBD2 (pdb code: 2KY8) are coloured green, blue and tan respectively.

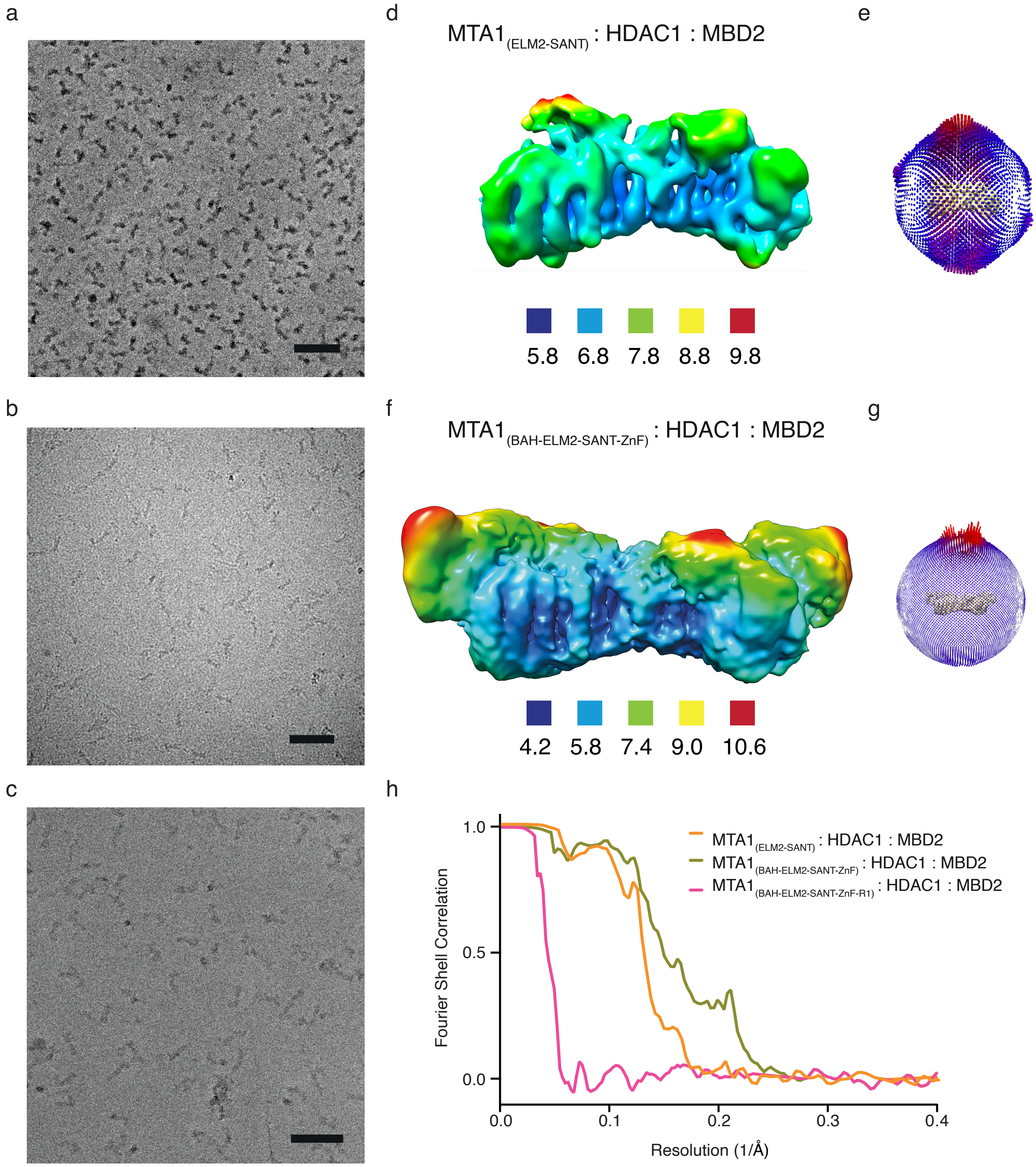

Supplementary Figure S3. Raw electron micrographs of the complexes and resolution estimates. (a) and (b) and (c) Representative electron micrograph of each of the MTA1 (residues 162-354), MTA1 (residues 1-453) and MTA1 (residues 1-546) NuRD complexes respectively. (d) and (e) Local resolution map of the MTA1 (residues 162-354) NuRD complex and the angular distribution of the final particle set. (f) and (g) Local resolution map of the MTA1 (residues 1-453) NuRD complex and the angular distribution of the final particle set. (h) Fourier Shell Correlation between the half maps for the three structures.

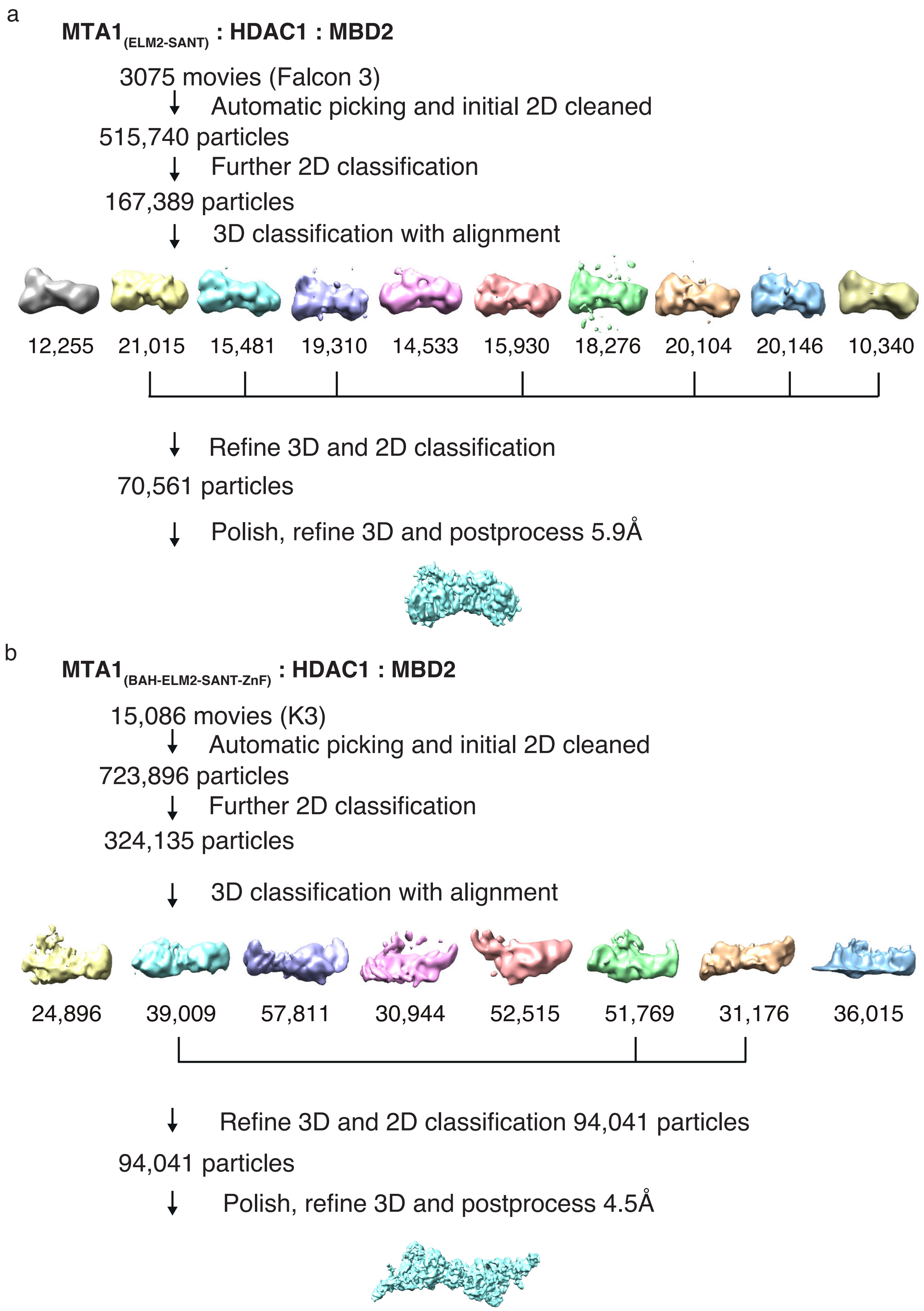

Supplementary Figure S4. Summary of the electron microscopy data processing steps in Relion3.0 for: (a) the complex containing MTA1 construct (residues 162-354); (b) the complex containing MTA1 construct (residues 1-453).

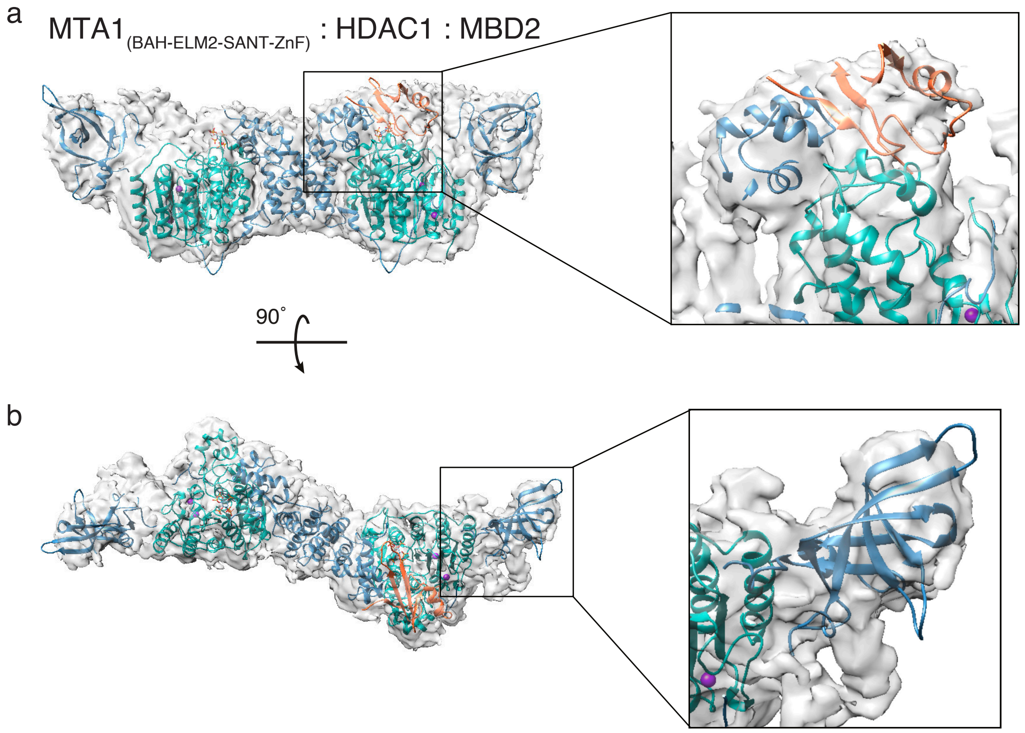

Supplementary Figure S5. Cryo-EM map showing the arrangement of the subunits with enlarged regions showing (a) the MBD domain of MBD2 (pdb code 2KY8) and (b) a phyre model of the BAH domain of MTA1 fitted into the map. HDAC1, MTA1 and MBD2 are coloured green, blue and tan respectively.

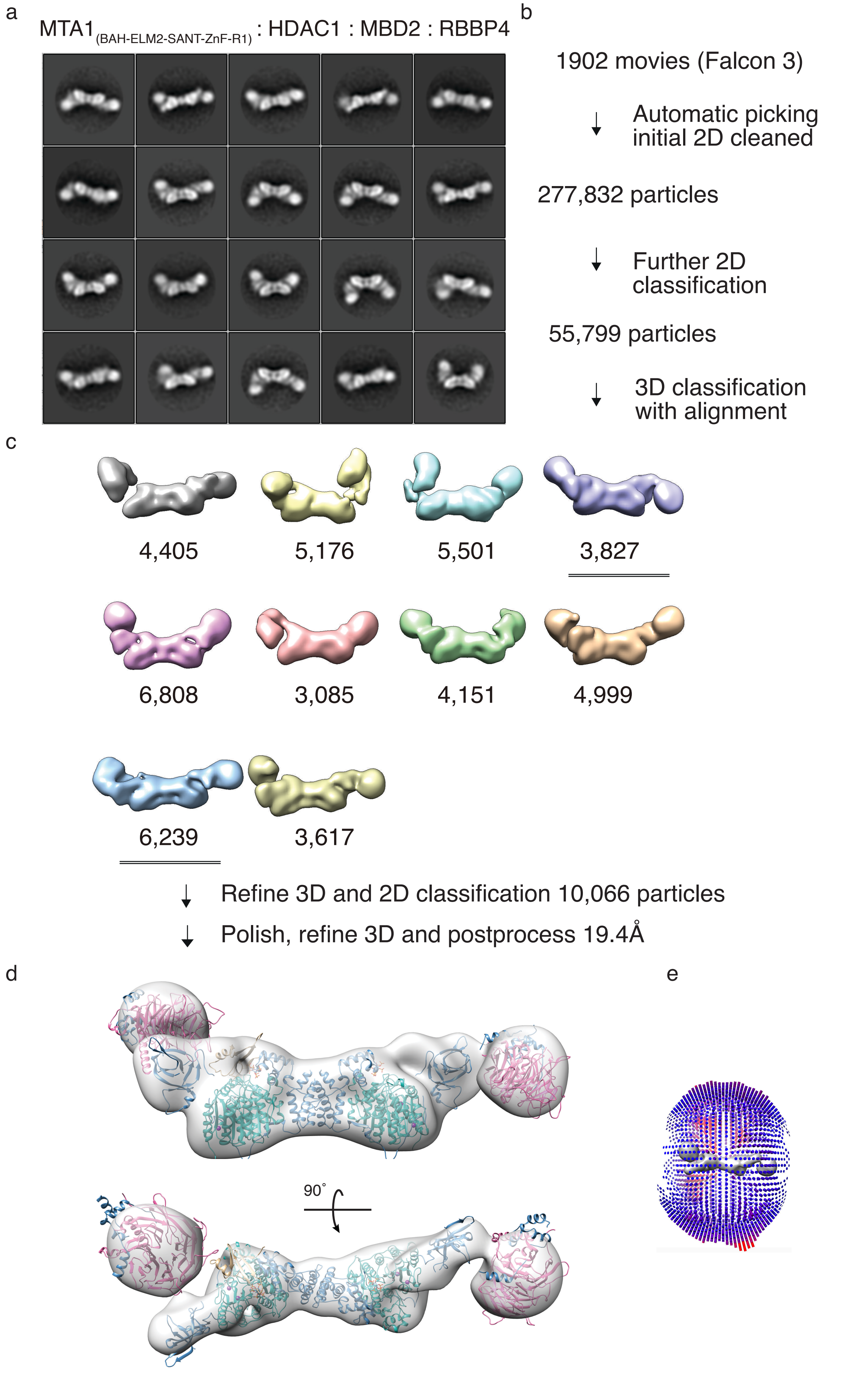

Supplementary Figure S6. The extended complex with RBBP4 shows structural flexibility. (a) Reference-free 2D class averages of the complex containing MTA1 construct (residues 1-546). (b) Electron microscopy data processing steps in Relion3.0. (c) 3D classification of particles into 10 classes. (d) Final 3D map of the complex calculated after combining two 3D classes and refining against 10,066 particles. The NMR solution structure (pdb code 2KY8) and crystal structures (pdb code 5ICN and 5FXY) are fitted into the map. (e) The angular distribution of the final particle set.
